# A Nest of LTR Retrotransposons Adjacent the Disease Resistance-Priming Gene *NPR1* in *Beta vulgaris* L. U.S. Hybrid H20

**DOI:** 10.1155/2009/576742

**Published:** 2009-04-15

**Authors:** David Kuykendall, Jonathan Shao, Kenneth Trimmer

**Affiliations:** Molecular Plant Pathology Laboratory, ARS, USDA, Beltsville, MD 20705, USA

## Abstract

A nest of long terminal repeat (LTR) retrotransposons (RTRs), discovered by LTR_STRUC analysis, is near core genes encoding the NPR1 disease resistance-activating factor and a heat-shock-factor-(HSF-) like protein in sugarbeet hybrid US H20. *SCHULTE*, a 10 833 bp LTR retrotransposon, with 1372 bp LTRs that are 0.7% divergent, has two ORFs with unexpected introns but encoding a reverse transcriptase with *rve* and *Rvt2* domains similar to *Ty1/copia*-type retrotransposons and a hypothetical protein. *SCHULTE* produced significant nucleotide BLAST alignments with repeat DNA elements from all four families of plants represented in the TIGR plant repeat database (PRD); the best nucleotide sequence alignment was to *ToRTL1* in *Lycopersicon esculentum*. A second sugarbeet LTR retrotransposon, *SCHMIDT*, 11 565 bp in length, has 2561 bp LTRs that share 100% identity with each other and share 98-99% nucleotide sequence identity over 10% of their length with DRVs, a family of highly repetitive, relatively small DNA sequences that are widely dispersed over the sugarbeet genome. *SCHMIDT* encodes a complete *gypsy*-like polyprotein in a single ORF. Analysis using LTR_STRUC of an in silico deletion of both of the above two LTR retrotransposons found that *SCHULTE* and *SCHMIDT* had inserted within an older LTR retrotransposon, resulting in a nest that is only about 10 Kb upstream of *NPR1* in sugarbeet hybrid US H20.

## 1. Introduction

Retrotransposons are now recognized as movers and shapers of plant genome evolution (see reviews [1, 2]). That retrotransposon elements account for much of the sugarbeet (*Beta vulgaris* L.) genome was shown by the identification [3] of repetitive DNA sequences in *Beta vulgaris* similar to long interspersed nuclear elements (LINEs), a type of retrotransposon without long terminal repeats (LTRs), and other repetitive DNA sequences that resembled LTR retrotransposons of the *Ty1-copia* class. A repeated DNA sequence in *Beta procumbens* was described as “*Athila*-like” [4] since it was deduced to be part of a long terminal repeat with similarity to the *Athila* retrotransposon from *Arabidopsis*.

Prior to the present study, pDRV sequences [5] were known simply as a family of short highly amplified DNA repeats shown by fluorescent in situ hybridization (FISH) technique to be widely dispersed over all 18 chromosomes of sugarbeet.

*Vulmar1,* a mariner-class DNA transposon in *Beta vulgaris* [6], is 3909 bp, has 32 bp terminal inverted repeats, and encodes, in a single ORF, a transposase with a characteristic “DDE” signature motif. Polymerase chain reaction (PCR) and fluorescent in situ hybridization (FISH) were used [6] to identify and to establish an abundance of *En/Spm*-like transposons in sugarbeet.

*Coe1*, a DNA transposon within apparent LTRs and other retrotransposon-like features, was discovered on a sugarbeet genomic BAC carrying the *NPR1* disease resistance-priming gene [7–9]. This recent discovery in *Beta vulgaris* of a unique 16.3 Kb CACTA *En/Spm*-like transposon named *Coe1* [7] was followed by the finding of conserved microsynteny of *NPR1* with another core plant gene whose predicted product has high similarity to a DNA-binding HSF protein [8]. About 70 Kb of repetitive DNA separates the *HSF* gene and *NPR1* from another small core gene cluster with a *CaMP* gene specifying a signal peptide calmodulin-binding protein and a gene encoding a CK1-class protein kinase gene [8], greatly extending and disambiguating the results of the initial sequencing and partial in silico analysis of an *NPR1* gene-carrying sugarbeet BAC [9]. In summary, our laboratory has identified, sequenced, and annotated a bacterial artificial chromosome (BAC) carrying the *NPR1* disease resistance priming gene of sugarbeet, *Beta vulgaris* L. [7–9].

Class I transposable elements which use reverse transcriptase to transpose via an RNA intermediate are termed retrotransposons. In order to identify possible LTR retrotransposons with LTRs, an intergenic region of repetitive DNA was examined by LTR_STRUC analysis, and this report details the discovery of a nest of retrotransposons about 10 Kb upstream from the *NPR1* disease resistance gene in sugarbeet H20. This nest appears to have formed when both a *copia*-type and a *gypsy*-type elements inserted within an older LTR retrotransposon. Two full-length sugarbeet LTR retrotransposons are described herein for the first time.

## 2. Materials and Methods

Identification of a sugarbeet BAC carrying the *NPR1* disease resistance control gene was described [9]. Genbank accession DQ851167 represents a partial sequence; the 38.6 Kb segment was the largest contig at that time. Subsequently the entire 130 Kb contiguous fragment was sequenced and annotated (Genbank accession EF101866). Basic methods used for DNA sequence analysis were described [9], and construction of the BAC library was detailed [10]. In the present study, LTR analyses of the *NPR1* BAC were performed using LTR_STRUC [11], and LTR Finder [12]. Programs, einverted [13] (http://bioweb.pasteur.fr/seqanal/interfaces/einverted), and EMBOSS (http://emboss.sourceforge.net/) [13] were used to identify inverted repeats, and repeats were also found using NCBI BLAST (http://www.ncbi.nlm.nih.gov/BLAST/). An EST database for sugarbeet (http://genomics.msu.edu/sugarbeet/blast.html) was employed for both nucleotide and protein BLAST to explore possible functional gene expression [14]. Subsequent analysis of DNA sequence data was performed using Lasergene version 6 (DNASTAR, Madison, Wis, USA). BLAST was used to identify the most similar protein products of LTR retrotransposons in other plant species. Multiple alignments were performed using MegAlign from the DNASTAR suite. Neighbor joining tree, or cluster analysis, was performed using MEGA 4 software (http://www.megasoftware.net/).

## 3. Results and Discussion

**Figure 1:**
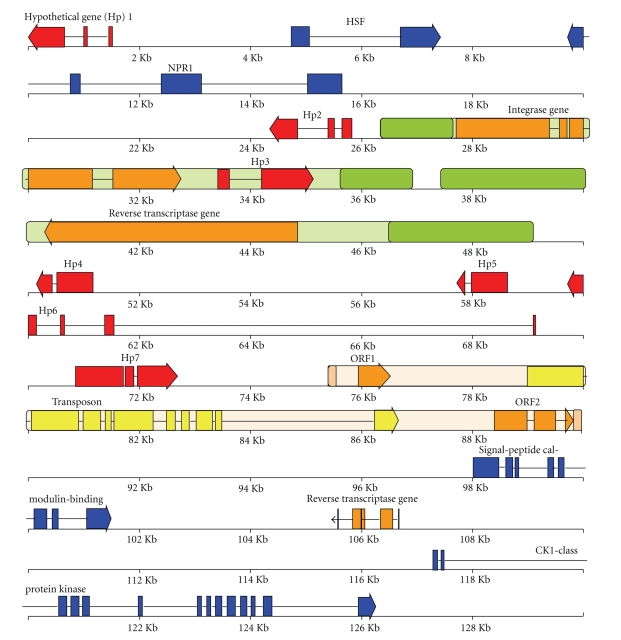
BAC physical map showing two LTR retrotransposons *SCHULTE* and *SCHMIDT* both highlighted in green and with darker green LTRs with rounded corners. On the other hand, the previously reported CACTA transposon *Coe1*, highlighted in light beige, has short tan LTRs, a yellow DNA transposase gene and orange ORF1 and ORF2. Exons are rectangles, and direction of arrows indicates direction of transcription. Exons of core plant genes are blue, and exons of hypothetical protein genes are red. Solid lines between exons depict introns. Scale is in 2 Kb increments. BAC is about 130 kilobase pairs. *SCHULTE* has an integrase gene in orange and a hypothetical gene in red. *SCHMIDT* has a single ORF retroelement reverse transcriptase polyprotein gene in orange.

A genomic *NPR1* disease resistance priming gene-carrying BAC [7–9] was subjected to LTR_STRUC and LTR FINDER analyses, and two distinct full-length LTR retrotransposons were identified (Figure 1). Depicted are *RTR1* and *RTR2,* two LTR retrotransposons that we also term *SCHULTE* and *SCHMIDT,* respectively, as well as a previously described element, *Coe1*, a DNA transposase gene within apparent LTRs and other retrotransposon-like features [7]. These repetitive DNA elements are intergenic, between two small clusters of core genes: *HSF* and *NPR1* genes separated from *CaMP* and *CK1PK* genes encoding a signal peptide calmodulin-binding protein and a “casein kinase 1-class protein kinase,” respectively.

**Figure 2:**
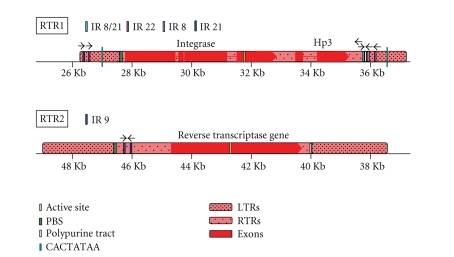
A detailed schematic of *Beta vulgaris* retrotransposons *SCHULTE* and SCHMIDT in red. Various numbered inverted repeat (IR) sequences in either light or dark blue or violet have arrows indicating relative direction. Green lines show the location of the DNA sequence motif CACTATAA. Heavily dotted ovular regions depict the size and position of the LTRs. The lightly dotted region shows the size of the whole retroelement. A green box shows the location of the polymerase binding site. Boxes show the position and size of exons. Lines between exons indicate introns. A centrally located small yellow box depicts the active site of the retroelement. An orange box shows the location of the polypurine tract. Scale is in Kb and is located underneath the illustrations in increments of 2 Kb per tick. Names of putative genes are located above the illustrations.

*SCHULTE,* a 10 833 bp long LTR retrotransposon, has 1372 bp LTRs sharing only 99.3% nucleotide sequence identity. The 0.7% divergence in the LTRs of *SCHULTE* indicates about ten base substitutions occurred since insertion/transposition. This old, somewhat degraded retrotransposon has two ORFs encoding a *Ty1/copia*-like integrase/reverse transcriptase and a hypothetical protein (Figure 2). Unexpected introns, uncharacteristic of retrotransposon genes, may be the result of frameshifts and point mutations. *SCHULTE* has 98% nucleotide sequence identity over ≥9 Kb with a 9.7 Kb DNA fragment (DQ374026) and 1.3 Kb of a 5.3 Kb DNA fragment (DQ374025), each fragment of BAC62 [14]. BAC62 carries a *Beta vulgaris* L. genomic region adjacent a *Beta procumbens* translocation carrying a nematode resistance gene [15], thus BAC62 has a *SCHULTE*-like retrotransposon.

Named to honor an author of the first-described physical map of the afore-mentioned region, *SCHULTE* is the first full-length retrotransposon sequence from *Beta vulgaris* to be reported. Since two out of the three *B. vulgaris* BACs sequenced to date, BAC62 and the *NPR1*-carrying BAC, carry a *SCHULTE*-like element, there are likely a very large number of *SCHULTE*-like LTR retrotransposons in the sugarbeet genome. However, *FLC*, or the flowering control gene-carrying BAC [16], did not carry a *SCHULTE*-like element.

**Figure 3:**
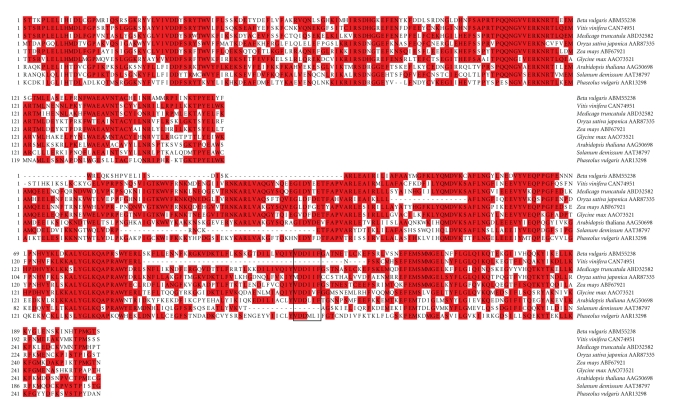
Amino acid residue alignment of *SCHULTE*'s integrase (top) and reverse transcriptase (bottom) domains. The active site of the RT domain from *Beta vulgaris* is boxed in along with corresponding sections of other reverse transcriptase proteins from different plants. Amino acids matching the consensus sequence are shaded. Numbers indicate cumulative amino acid positions; *Arabidopsis thaliana* (AAG50698), *Beta vulgaris* (ABM55238), *Glycine max* (AAO73521), *Medicago truncatula* (ABD32582), *Oryza sativa* japonica (AAR87335), *Phaseolus vulgaris* (AAR13298), *Solanum demissum* (AAT38797), *Vitis vinifera* (CAN74951), *Zea mays* (ABF67921).

**Figure 4:**
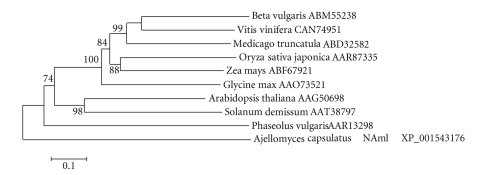
Similarity tree constructed by neighbor joining method of the reverse transcriptase domain of *SCHULTE* from *Beta vulgaris* along with corresponding domains of other reverse transcriptase proteins from different plants. The numbers on the branches are bootstrap (confidence) values. Genbank accession numbers of amino acid sequences are given following plant names.

SMART analysis showed that the predicted product of the *SCHULTE* reverse transcriptase gene has *rve* and *Rvt2* protein domains. An alignment by MegAlign of the conserved *rve* and *Rvt2* (Figure 3) domains of similar *Ty1-copia*-like plant retrotransposon-encoded proteins, identified by BLAST, were analyzed by neighbor joining in MEGA 4 to assess structural relatedness (Figure 4). As shown in Figure 3, the predicted product of the *Beta vulgaris SCHULTE* reverse transcriptase gene has conserved *rve* and *Rvt2* domains shared among highly similar domains of products of LTR retrotransposons from *Medicago truncatula*, *Vitis vinifera, Oryza sativa japonica*, *Zea mays,* and *Glycine max*. Except for the *Solanum demissum* and *Vitis vinifera* accessions, the *Rvt2* domain evidently has a conserved YVDDIIF active site (Figure 3).

Similar LTR retrotransposon gene products in *Arabidopsis thaliana*, *Solanum demissum*, and particularly in *Phaseolus vulgaris* are structurally divergent (Figures 3 and 4). A search of the TIGR plant repeat database revealed that *SCHULTE* produced nucleotide sequence matches with many different *copia*-like retrotransposons in all four families: Brassicaceae, Fabaceae, Gramineae, and Solanaceae. The best PRD nucleotide sequence alignment match (*E* = 3.8e^−232^) was to *ToRTL1* in *Lycopersicon esculentum*.

Probable expression of integrase/reverse transcriptase gene(s) in active *SCHULTE*-like retrotransposon was shown by BLAST alignment (*E* = 0.0) of the ORF with BI643218, an EST, or expressed sequence tag. Expression of both LTRs was clearly evidenced by alignments, *E* = 0.0, with ESTs BI698297 and BI698341. Four other ESTs showing some alignment (*E* = e^−151^ to *E* = 9e^−36^) also suggest other likely active *SCHULTE*-like elements.

Another LTR retrotransposon discovered using LTR_STRUC and LTR FINDER, *SCHMIDT* was so named to honor a pioneering researcher of repeat DNA elements in *Beta*. *SCHMIDT*, 11 565 bp retroelement, encodes a complete *Ty3-gypsy*-class polyprotein in a single ORF without introns. The *SCHMIDT* reverse transcriptase gene has all of the domains expected of an intact retroelement polyprotein, and the domain order is indicative of *Ty3-gypsy*-class. *SCHMIDT* has 2561 bp LTR sequences with 100% identity, consistent with this transposable element still being active.

EMBOSS analysis of the 130 Kb *NPR1*-carrying sugarbeet BAC revealed the presence of at least 24 inverted repeat (IR) sequences but, for the purposes of this report, let us describe only those inverted sequences associated with LTR retrotransposons *SCHULTE* and *SCHMIDT*. The following two pairs of *IR 8/21* inverted repeat sequences are associated with *SCHULTE* (Figure 2). The *IR 8/21* inverted repeat sequences share 94% identity (18/19). 
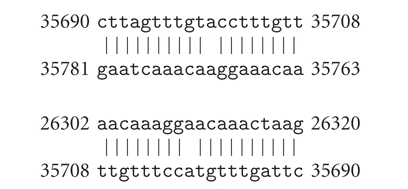
 The following pair of *IR 22* inverted sequences, associated with *SCHULTE* (Figure 2), are 9.5 Kb apart and share 80% identity (shown below), but this pair of *IR 22* are also direct repeats with 96% identity. 
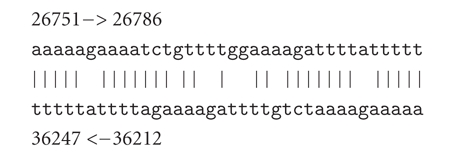

*SCHMIDT* has a pair of inverted repeat *IR 9* sequences upstream of the single reverse transcriptase polyprotein gene and just downstream of polymerase binding site (Figure 2). These *IR 9* inverted repeat sequences share 100% identity (23/23) and contain only nucleotides A and T.

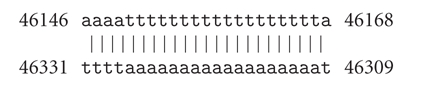



Over about one-tenth of their length, each LTR sequence of *SCHMIDT* shares 97% nucleic acid sequence identity with *pDRV*, a family of *Beta vulgaris* short repeated DNA sequences known as rich in *DraI* (TTTAAA) restriction enzyme recognition sites [5]. For example, eight *DraI* sites are carried by *pDRV1,* a 434 bp repeated sequence [5]. Each LTR of *SCHMIDT* has 10 *DraI* sites, and the full-length *SCHMIDT* retrotransposon has twenty-four *DraI* (TTTAAA) sites. Perhaps *pDVR* conserved sequences were originally a preferred recognition site for insertion; they seem to have evolved into an integral part of the LTRs of *SCHMIDT*-like retrotransposons. In any case, the observed high degree of nucleotide sequence identity that the LTRs of *SCHMIDT* have with pDRVs, highly reiterated sequences rich in nucleotides A and T [5], is very interesting since *pDRV* repeat sequences, originally visualized by FISH, are dispersed over all 18 sugarbeet chromosomes [5].

**Figure 5:**
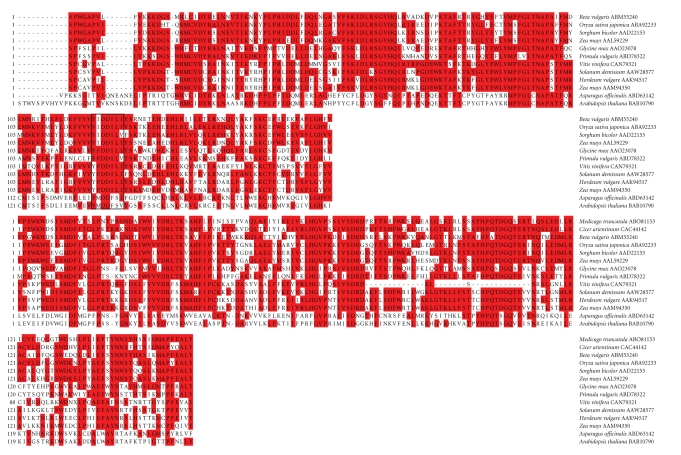
Amino acid residue alignment of the reverse transcriptase (top) and integrase (bottom) domains of *SCHMIDT* from *Beta vulgaris*, with its active site boxed in, along with corresponding sections of other reverse transcriptase proteins from different plants. Amino acids matching the consensus sequence are shaded. Numbers indicate cumulative amino acid positions; *Arabidopsis thaliana* (BAB10790), *Asparagus officinalis* (ABD63142), *Beta vulgaris* (ABM55240), *Cicer arietinum* (CAC44142), *Glycine max* (AAO23078), *Hordeum vulgare* (AAK94517), *Medicago truncatula* (ABO81153), *Oryza sativa* japonica (ABA92233), *Primula vulgaris* (ABD78322), *Solanum demissum* (AAW28577), *Sorghum bicolor* (AAD22153), *Vitis vinifera* (CAN79321), *Zea mays* (AAM94350), *Zea mays* (AAL59229).

**Figure 6:**
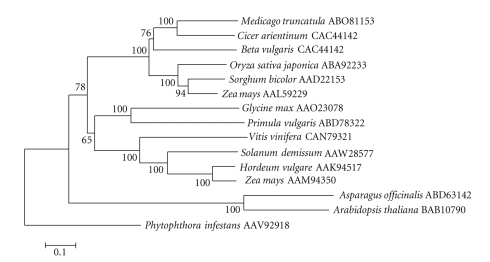
Similarity tree constructed by neighbor joining method of the reverse transcriptase domain of *SCHMIDT* from *Beta vulgaris* along with corresponding domains of other reverse transcriptase proteins from different plants. The numbers on the branches are bootstrap (confidence) values. Genbank accession numbers of amino acid sequences are given following plant names.

Figure 5 shows results of alignment by MegAlign of conserved *rve* and *Rvt1* domains of the protein products from *SCHMIDT*-like plant retrotransposons identified by BLASTp from various plant species, and Figure 6 shows a neighbor joining analysis tree by MEGA 4 showing structural relatedness. A highly conserved FIDDILI active site in the *Rvt1* domain is noted in particular (Figure 5). The predicted *SCHMIDT* reverse transcriptase polyprotein gene product shows high structural similarity with a conserved region of proteins encoded by similar LTR retrotransposons from *Cicer arietinum, Medicago truncatula, Oryza sativa japonica, Sorghum bicolor*, and *Zea mays* AAL59229. Somewhat similar LTR retrotransposons from *Glycine max*, *Primula vulgaris, Vitis vinifera, Solanum demissum*, *Hordeum vulgare*, and *Zea mays* AAM94350 produce structurally divergent products (Figure 6). Against the TIGR plant repeat database, *SCHMIDT* produced good, *E* ≤ e^−50^, BLAST nucleotide sequence identity which matches primarily with *Prem1*- and *Xilon1*-like *gypsy*-like RTRs in *Zea*, *Oryza*, *Sorghum*, and *Triticum*. This finding suggests divergent evolution, where a *SCHMIDT*-like ancestor originated in monocots, then, upon lateral transfer to sugarbeet, natural selection for structural similarity or convergence in a new genetic background resulted in a high degree of amino acid similarity of the protein product with other *gypsy*-like retrotransposons in eudicots. The predictions of convergent evolution are structurally similar proteins encoded by phylogenetically distinct retrotransposons. Whether similar sequences arose through convergent or divergent evolution, it is interesting to simply note that *SCHMIDT* has a significant degree of nucleotide sequence identity primarily with certain *gypsy*-like retrotransposons found in monocots.

Expression of retroelements similar to SCHMIDT in sugarbeets is suggested by the finding that *SCHMIDT* gave BLAST alignments with the following ESTs: BI643170, BI643158, BI698360, and BI643246 (*E* = 10^−161^, 3*E* = 10^−79^, *E* = 10^−68^, and 5*E* = 10^−59^, resp.). These four BLAST hits represent only about 0.02% of the ESTs in the collection.

An older LTR retrotransposon, which had been interrupted by subsequent insertions of *SCHULTE* and *SCHMIDT*, became evident (Table 1) when LTR_STRUC analysis was performed on a sequence having an in silico deletion of the LTR retrotransposons *SCHULTE* and *SCHMIDT*. Although very degraded and unclassifiable, the older LTR retrotransposon was deduced to be 5395 bp with 780 bp LTRs sharing 99% identity.

In conclusion, the relatively small repetitive DNA sequences previously described as “*pDRV*s” can now be seen as a part of the LTRs of *SCHMIDT*-like retrotransposons.

Planned research will address possible effects of retrotransposons on the expression of core plant genes including the *NPR1* disease resistance-priming gene immediately downstream of the LTR retrotransposon nest.

## 4. Conclusions

An LTR retrotransposon nest consisting of an older retroelement into which both a *gypsy*-like *SCHMIDT* and a *copia*-like *SCHULTE* inserted was identified, and properties of the retrotransposons were described. Since LTR retrotransposons are driving forces in plant genome evolution (see reviews [1, 2]), they may have tremendous potential usefulness in genetic manipulation and genome modification to enhance agricultural profitability and sustainability.

## Figures and Tables

**Table 1:** Several LTR retrotransposons discovered within a TE nest, in addition to *Coe1* [7], by LTR_STRUC analysis of a sugarbeet genomic BAC carrying *NPR1*.

Feature	*SCHULTE*	*SCHMIDT*	*Older LTR-RTR*	*Coe1* [7]
Active site	YVDDIIL	FIDDILI	SCDDVLL	YVDDIIL
Length of RTR	10 833 bp	11 565 bp	5395 bp (before two TE insertions)	14 531 bp
Length of LTR	1372 bp	2561 bp	780 bp	169 bp
5′ LTR-3′ LTR Identity (%)	99.3%	100.0%	99.0%	96.4%
Number of open reading frames (ORFs)	2	1	2	3
5′ beginning and 3′ end of flanking region (duplication)	ATTTT	CGCTC	GCTTG	CTACT
Class & domains present	*Copia*-like Integrase and RTase domains	All domains expected of a complete *Gypsy*-like retrotransposon	Putative RT domains	*Coe1* [7], class II within a class I, DNA transposase/*Copia*-like RTase pseudogene
